# Attitudinal openness to AI as supervisor or colleague/subordinate in Japan: the role of advanced digital technology familiarity

**DOI:** 10.3389/frai.2026.1920376

**Published:** 2026-08-18

**Authors:** Makoto Nakakita, Takahiro Hoshino

**Affiliations:** 1 Center for Advanced Intelligence Project, RIKEN, Tokyo, Japan; 2 Faculty of Economics, Keio University, Tokyo, Japan

**Keywords:** advanced digital technology familiarity, AI as supervisor, AI in business, employee attitudes, hierarchical Bayesian panel model, Japan, workplace AI role openness

## Abstract

Organizations adopting artificial intelligence (AI) must account for employees' attitudes toward AI in different workplace roles. This study examines whether familiarity with advanced digital technologies is associated with openness to AI as a supervisor or as a colleague/subordinate in Japan. We analyze a three-wave balanced panel of continuously employed respondents using hierarchical Bayesian panel models. The focal measure is the Advanced Digital Technology Familiarity Index (ADTFI), based on self-reported familiarity with six technologies; the outcome is stated openness to hypothetical AI role arrangements rather than realized adoption. Higher ADTFI is positively but modestly associated with workplace AI role openness. A covariate-adjusted coefficient decomposition shows that computing capability and workplace technology exposure have positive ADTFI-related components, while workplace technology exposure has an imprecisely estimated negative conditional component and a combined component whose interval includes zero. Role-specific ordered-probit models yield a negative TechAdop coefficient with an interval excluding zero for the AI-supervisor item, whereas the corresponding colleague/subordinate estimate is near zero and imprecise; this descriptive contrast is not a formal cross-role coefficient test. A lagged model retains a small positive association between prior ADTFI and subsequent openness after prior openness is controlled. The results mainly reflect stable between-person differences and are conditional on continuously employed panel respondents. They should therefore be interpreted as descriptive association patterns, not causal effects of training or AI implementation.

## 1 Introduction

As artificial intelligence (AI) systems enter organizational decision support, workflow coordination, and human–AI collaboration, firms confront not only technical implementation questions but also variation in employee attitudes. The role assigned to AI matters: accepting AI as a tool or colleague is not equivalent to accepting it as a supervisor with authority over evaluation, coordination, or task allocation (Jarrahi, 2018; Raisch and Krakowski, 2021; Longoni et al., 2019). This study examines one potential antecedent of such variation: employees' familiarity with advanced digital technologies.

The focal measure is deliberately defined as familiarity rather than objective capability. It records self-assessed knowledge of IoT, cloud computing, AI, RPA, machine learning, and deep learning. This type of familiarity may reduce perceived opacity and make AI-enabled workflows easier to understand, but it can also reflect prior exposure, confidence, occupation, and other stable characteristics. Computing-task capability is therefore retained as a distinct covariate rather than treated as synonymous with the familiarity index. Because both ADTFI and AIAccept are self-reports, their association may also reflect common-method variance, social desirability, overconfidence, anchoring, or stable response styles.

We use three waves of Japanese household-panel data to examine four questions. First, how strongly is advanced digital technology familiarity associated with stated openness to workplace AI roles? Second, which demographic, occupational, workplace, and household characteristics are associated with familiarity? Third, how do covariate associations with AI-role openness decompose algebraically into an Advanced Digital Technology Familiarity Index (ADTFI)-related component and a component conditional on ADTFI? Fourth, do the associations differ between AI as supervisor and AI as colleague/subordinate?

The study contributes to AI in Business research in three ways. It provides employee-level panel evidence on attitudes toward role-configured workplace AI; it connects digital-inequality research with organizational AI attitudes while avoiding claims that familiarity is an objective skill test; and it uses hierarchical Bayesian models, a lagged specification, a within–between sensitivity analysis, and role-specific ordinal models to characterize uncertainty and scope. The analysis is observational. Its main contribution is therefore descriptive and explanatory rather than causal.

The remainder of the paper reviews the relevant literature, describes the data and measures, presents the analytical framework and results, and concludes with theoretical, managerial, and ethical implications.

## 2 Theoretical background and research questions

### 2.1 Digital inequality and technology familiarity

Digital inequality extends beyond access to differences in skills, use, confidence, and outcomes (Hargittai, 2002; van Dijk, 2005; Robinson et al., 2015; Scheerder et al., 2017). Familiarity with advanced technologies is one subjective component of this broader landscape. It may be patterned by age, occupation, workplace exposure, and household resources, yet it is not equivalent to demonstrated task performance. The present paper therefore distinguishes ADTFI from the Computing Skill Index, which records whether respondents can perform spreadsheet, macro, and programming-related tasks.

### 2.2 Workplace AI roles, algorithmic authority, and acceptance

People do not respond uniformly to algorithmic systems. Algorithmic-aversion and algorithm-appreciation research shows that reactions depend on perceived error, task context, control, and the authority assigned to the system (Dietvorst et al., 2015; Logg et al., 2019; Castelo et al., 2019). Trust, explainability, fairness, and legitimacy may also shape acceptance, although these constructs are not directly measured here (Afroogh et al., 2024; Longoni et al., 2019). TAM and UTAUT identify usefulness, effort, social influence, and facilitating conditions as relevant to technology use (Davis, 1989; Venkatesh et al., 2003). ADTFI is best viewed as an upstream familiarity measure that may correlate with employee-side facilitating conditions, while TechAdop captures workplace exposure to current or planned technologies.

Acceptance, trust, adoption, and readiness are kept conceptually separate. AIAccept measures stated resistance or openness to hypothetical workplace role configurations. It does not measure trust in AI, behavioral intention to use a particular system, realized adoption, or firm-level readiness. Moreover, the supervisor item and the colleague/subordinate item combine distinct authority and collaboration relationships, so the combined index is supplemented by role-specific models.

### 2.3 Research questions

RQ1. How is advanced digital technology familiarity associated with stated openness to workplace AI roles?

RQ2. Which demographic, occupational, workplace, and household characteristics are associated with advanced digital technology familiarity?

RQ3. How do selected covariate associations with workplace AI role openness decompose into an ADTFI-related component and a component conditional on ADTFI?

RQ4. Do focal associations differ between the AI-supervisor and AI colleague/subordinate scenarios?

## 3 Data and measures

The study uses the 2021–2023 Japan Household Panel Survey/Keio Household Panel Survey, administered by the Panel Data Research Center at Keio University. The harmonized file pools KHPS, JHPS, and a newer JHPS cohort. The initial file contains 13,507 person-wave records from 5,021 individuals. After residence and employment restrictions, the analytical panel contains 1,976 respondents observed in all three waves. Baseline models using the combined AIAccept index require post-harmonization values on both AI-role items, yielding 5,888 person-wave observations from 1,970 respondents. Sensitivity analyses that require both items to have been originally observed use 5,780 person-wave observations from 1,961 respondents. The estimand is therefore conditional on stable, continuously employed panel respondents and should not be generalized to the entire Japanese workforce. At the 2021 baseline, retained and excluded employed respondents differed modestly in income, sex composition, and computing capability; Supplementary Table S2 reports the comparison. An inverse-probability-weighted sensitivity based on observed baseline retention predictors preserves the positive ADTFI association, although it cannot address selection on unobserved factors (Supplementary Table S3).

The original harmonization script first recoded item-specific missing-value codes and then applied one-nearest-neighbor (1NN) imputation to the full 93-column harmonized matrix before the sample restrictions were imposed. It used one neighbor, scikit-learn's default nan-Euclidean distance, pooled donors across survey years and source panels, and did not restrict donors to otherwise similar respondents. Consequently, missing values in covariates and a small number of ADTFI and AIAccept items could be replaced; the procedure did not propagate imputation uncertainty. Because one observed donor supplied each replacement after item-specific recoding, imputed values remained on the observed support of the corresponding variable.

Using the raw wave files and the original harmonization code, we reconstructed the pre-imputation missingness patterns for the 5,928 person-wave records matching the baseline analytical sample. Raw missingness was 2.41% for the AI-supervisor item and 2.43% for the colleague/subordinate item; item-specific missingness ranged from 0.64% to 0.91% across the six ADTFI items. Missingness was higher for several economic covariates, including individual income (5.23%), household savings (7.05%), household assets (7.81%), and household income (9.35%). Supplementary Table S5 provides the audit summary, and Supplementary Table S4 distinguishes raw item availability from the post-harmonization outcome sample.

We additionally reconstructed a strict balanced complete-case sample requiring all Model 2 inputs to be observed in all three waves. This sensitivity sample contains 1,158 respondents and 3,474 person-wave observations. The focal ADTFI coefficient remains positive and similar in magnitude [0.115, 95% HPDI [0.045, 0.189]] to the baseline estimate [0.101, 95% HPDI [0.043, 0.157]]; the directions of the other focal associations are also broadly preserved (Supplementary Table S6).

We also conducted a formal multiple-imputation sensitivity analysis using the reconstructed pre-imputation panel. Twenty completed datasets were generated in person-wide form with stochastic chained equations using Bayesian-ridge conditional models and posterior predictive sampling, so values from other waves could inform a respondent's missing ADTFI items and covariates. Discrete variables were mapped back to their observed valid supports. AIAccept was not imputed; every outcome record retained in this sensitivity analysis had both raw AI-role items observed, yielding 5,780 person-wave observations from 1,961 respondents. Each completed dataset was analyzed with the Model 2 hierarchy using four chains, 3,000 warm-up iterations and 3,000 retained iterations per chain, and coefficient summaries were combined using Rubin's rules. The procedure is a pragmatic missing-data sensitivity analysis rather than a jointly estimated multilevel Bayesian missing-data model.

### 3.1 Advanced digital technology familiarity and workplace AI role openness

The Advanced Digital Technology Familiarity Index (ADTFI) is the reverse-coded mean of six five-point familiarity items covering IoT, cloud computing, AI, RPA, machine learning, and deep learning. Higher scores indicate greater self-reported familiarity. We use the label ADTFI rather than “digital capability” because the items do not objectively test performance and may be affected by self-presentation, overconfidence, or anchoring. An AI-neutral three-item version based on IoT, cloud, and RPA is reported prominently as a measurement sensitivity check.

AIAccept is the mean of two five-point items asking about resistance if AI became (1) the respondent's supervisor and (2) the respondent's colleague or subordinate. The combined index is calculated only when both post-harmonization item values are valid on the 1–5 scale; it is never calculated from a single valid item. Raw-observed sensitivity analyses require both items to have been observed before imputation, and raw-code zeros are treated as non-response. Higher values indicate greater openness. The outcome is an attitude toward hypothetical role relationships, not an observed adoption behavior.

### 3.2 Covariates

The models include year, standardized age and age squared, marital status, gender, cohabitation with younger household members, employment form, occupation, organization type, desire to quit, technology exposure, computing-task capability, wellbeing, health-related routines, log-transformed household resources, and region. TechAdop and Comput are rescaled to the unit interval. Clerical workers are the occupational reference group because the residual “other occupation” category contains only eight person-wave observations and produces unstable comparisons. Supplementary Table S8 reports exact category counts.

### 3.3 Descriptive statistics

**Figure 1:**
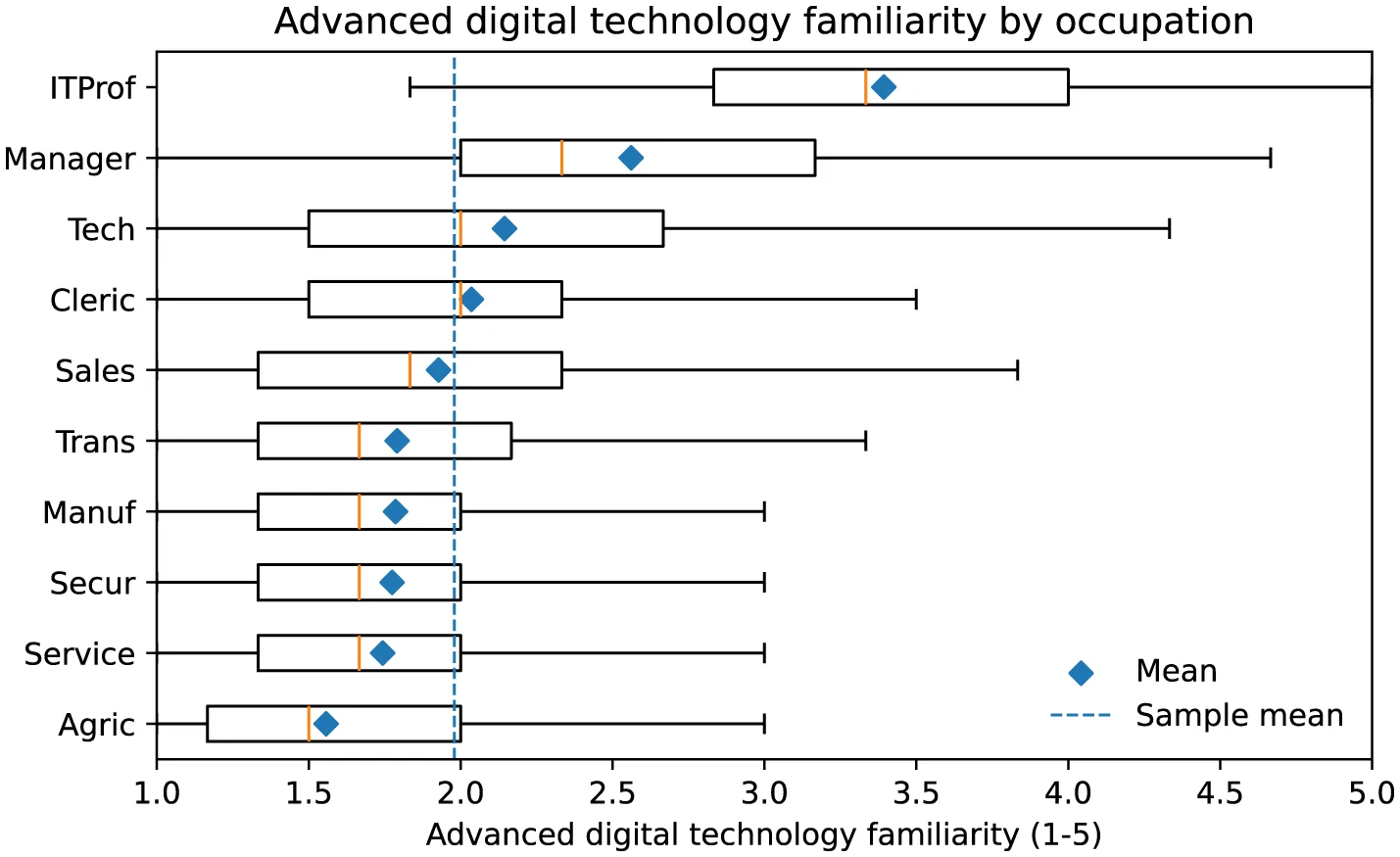
Distribution of ADTFI across the principal occupational categories. The small unclassified/other category is omitted from the graphic but retained as a coefficient in the models; clerical workers are the regression reference group.

Descriptive statistics for the analytical sample are reported in Table 1, and occupational differences in ADTFI are summarized in Figure 1.

**Table 1:** Descriptive statistics.

Variable	Description	Mean	SD	Max	Min
Focal variables					
ADTFI	Advanced digital technology familiarity index (1–5)	1.979	0.756	5.000	1.000
AIAccept	Openness to hypothetical workplace AI roles (1–5)	2.377	1.160	5.000	1.000
TechAdop	Workplace technology exposure (rescaled 0–1)	0.090	0.185	1.000	0.000
Comput	Computing task capability (rescaled 0–1)	0.290	0.333	1.000	0.000
Individual attributes					
Year22^†^	Year 2022	0.333	0.471	1.000	0.000
Year23^†^	Year 2023	0.333	0.471	1.000	0.000
Age	Age in years	52.525	12.672	89.000	23.000
AgeSq	Age squared	2919.449	1343.767	7921.000	529.000
Married^†^	Married	0.701	0.458	1.000	0.000
Male^†^	Male	0.553	0.497	1.000	0.000
Cohabit^†^	Living with younger household member(s)	0.488	0.500	1.000	0.000
WorkStu^†^	Working and studying	0.005	0.070	1.000	0.000
WorkHom^†^	Working and housework	0.147	0.354	1.000	0.000
Occupational indicators (reference: clerical workers)					
OtherOcc^†^	Unclassified, mining, or other occupation	0.001	0.037	1.000	0.000
Agric^†^	Agriculture/fishery	0.025	0.155	1.000	0.000
Sales^†^	Sales	0.144	0.351	1.000	0.000
Service^†^	Service	0.189	0.392	1.000	0.000
Manager^†^	Managerial	0.045	0.207	1.000	0.000
Trans^†^	Transport/communication	0.044	0.206	1.000	0.000
Manuf^†^	Manufacturing/construction	0.153	0.360	1.000	0.000
ITProf^†^	IT professional	0.028	0.165	1.000	0.000
Tech^†^	Professional/technical	0.169	0.374	1.000	0.000
Secur^†^	Security	0.016	0.126	1.000	0.000
Organization, controls, resources, and region					
Private^†^	Private corporation employee	0.553	0.497	1.000	0.000
NonProf^†^	Non-profit employee	0.130	0.336	1.000	0.000
Public^†^	Public-sector employee	0.079	0.270	1.000	0.000
QuitJob^†^	Wishes to quit current job	0.139	0.346	1.000	0.000
Wellbeing	Subjective wellbeing (0–10)	6.021	2.117	10.000	0.000
Alcohol^†^	Drinking habit	0.590	0.492	1.000	0.000
Smoking^†^	Smoking habit	0.184	0.388	1.000	0.000
Exercise^†^	Regular exercise	0.326	0.469	1.000	0.000
Income	Individual income, log(*x*+1)	14.621	1.838	17.786	0.000
Saving	Household savings, log(*x*+1)	11.381	6.971	19.859	0.000
Asset	Household assets, log(*x*+1)	3.077	6.080	18.603	0.000
HHIncome	Household income, log(*x*+1)	15.584	0.702	18.370	0.000
Urban^†^	Other city	0.586	0.493	1.000	0.000
Rural^†^	Town/village	0.084	0.277	1.000	0.000

^†^Binary variable. Age and AgeSq are shown on their raw scales but standardized in estimation. Baseline models using the combined AIAccept index require two post-harmonization role-item values; 5,888 person-wave observations from 1,970 respondents enter those models. Raw-item sensitivities require both items to have been originally observed. TechAdop and Comput are reported after rescaling to the unit interval.

## 4 Methods

### 4.1 Model specifications

Four hierarchical Bayesian panel specifications are reported. Model 1 regresses ADTFI on the covariates. Model 2 regresses AIAccept on ADTFI and the same covariates. Model 3 omits ADTFI and provides a reduced-form comparison. Model 4 predicts current AIAccept from prior-wave ADTFI and prior-wave AIAccept while retaining an individual random intercept. In Model 4, the posterior random-intercept scale is small after prior AIAccept is included, indicating little residual stable between-person heterogeneity. The role-specific analyses estimate the two ordinal items separately. The familiarity and outcome models are defined in Equations 1 and 2, and the descriptive decomposition is defined in Equations 3–5.

For individual *i* at wave *t*, the familiarity and outcome equations are


$$\begin{array}{ll} F_{it} & =\alpha_{i}^{\left(F\right)}+{x_{it}}^{⊤}\beta^{\left(F\right)}+\varepsilon_{it}^{\left(F\right)}, \end{array}$$ (1)

$$\begin{array}{ll} Y_{it} & =\alpha_{i}^{\left(Y\right)}+\gamma F_{it}+{x_{it}}^{⊤}\beta^{\left(Y\right)}+\varepsilon_{it}^{\left(Y\right)}. \end{array}$$ (2)

Here, *F*_*it*_ is ADTFI and *Y*_*it*_ is AIAccept. The equations are estimated separately, with equation-specific random intercepts. Their covariance is not estimated in the baseline specification. Consequently, the algebraic product $\gamma \beta_{j}^{\left(F\right)}$ is not interpreted as an identified causal indirect effect.

For descriptive decomposition, we report


$$\begin{array}{l} A_{j}^{\left(F\right)}=\gamma \beta_{j}^{\left(F\right)}\text{ (ADTFI-related component)}, \end{array}$$ (3)

$$\begin{array}{l} A_{j}^{\left(C\right)}=\beta_{j}^{\left(Y\right)}\text{ (conditional component)}, \end{array}$$ (4)

$$\begin{array}{l} A_{j}^{\left(T\right)}\;=A_{j}^{\left(F\right)}+A_{j}^{\left(C\right)}\text{ (combined component)}. \end{array}$$ (5)

The two equations were sampled independently. To summarize the product distribution, equal-sized simple random samples were drawn independently from the flattened posterior of each equation and paired one-to-one. This carries uncertainty from both equations into the reported interval under the maintained cross-equation independence assumption. Baseline slopes combine within- and between-person variation; a Mundlak-style sensitivity analysis separates those components and indicates that the focal familiarity–openness association is primarily between persons.

### 4.2 Estimation and diagnostics

All four panel specifications use normal likelihoods, individual random intercepts, weakly informative normal priors for slopes, and half-Cauchy priors for scale parameters (Polson and Scott, 2012). Continuous non-binary covariates are mean-centered, and four chains are used throughout. Model 1 uses 4,000 warm-up and 4,000 retained iterations per chain; Models 2 and 3 use 2,500/2,500 and 2,000/2,000 iterations, respectively; and Model 4 uses 3,000/3,000 iterations. An Ancillarity–Sufficiency Interweaving Strategy was used for the lagged model to stabilize sampling of the small random-intercept scale (Yu and Meng, 2011). Convergence is assessed with $\hat{R}$, bulk and tail effective sample sizes, Monte Carlo standard errors, and trace inspection. Across the four final production models, the maximum $\hat{R}$ is 1.006, the minimum bulk effective sample size is 1,101, and the minimum tail effective sample size is 1,906. Supplementary Table S12 reports model-level diagnostic summaries, and Supplementary Section S1 provides the sampling details.

For the formal multiple-imputation sensitivity, each of the 20 completed datasets was fitted with four chains, 3,000 warm-up iterations, and 3,000 retained iterations per chain. Diagnostics were assessed separately within every completed dataset. Across all parameters and imputations, the maximum $\hat{R}$ was 1.005, the minimum bulk effective sample size was 1,581, the minimum tail effective sample size was 3,344, and the largest Monte Carlo standard error of a posterior mean was 0.0058.

Because ADTFI and AIAccept are bounded and discrete, Supplementary Table S13 reports posterior-predictive calibration for the Gaussian index models. We retain the Gaussian index models because the principal estimand is a covariate-adjusted conditional mean of an averaged index, and the linear form permits transparent coefficient decomposition and direct comparison of Models 2 and 3. This choice does not assume that AIAccept is unbounded or normally distributed. The check shows that 7.6% of ADTFI predictions and 13.1% of marginal AIAccept predictions fall outside the 1–5 scale. The 13.1% rate is substantial enough that the Gaussian AIAccept likelihood cannot be regarded as an adequate model of the full bounded response distribution. Accordingly, the Gaussian results are interpreted only as covariate-adjusted conditional-mean associations, not as estimates of category probabilities or tail behavior. We therefore treat the nine-category cumulative-probit model for the combined AIAccept index and the Bayesian ordered-probit models for the two role items as essential functional-form sensitivities. These ordinal analyses provide distributionally appropriate evidence on the focal sign pattern but do not reproduce or fully validate the hierarchical Gaussian likelihood. WAIC comparisons are not reported because effective-parameter estimates were unstable and models using different samples are not directly comparable.

## 5 Results

### 5.1 Advanced digital technology familiarity

ADTFI declines with age and is higher among men, managers, IT professionals, professional/technical workers, private-sector employees, respondents with greater workplace technology exposure and computing-task capability, and respondents with greater household resources. Relative to clerical workers, agriculture, transport/communication, and manufacturing/construction workers have lower ADTFI, while the prior large positive coefficients for nearly every occupation disappear after replacing the eight-observation residual reference group.

### 5.2 Workplace AI role openness with and without ADTFI

In Model 2, ADTFI has a positive but modest conditional association with AIAccept [0.101, 95% HPDI [0.043, 0.157]]. Male, WorkHom, QuitJob, and a convex age term are positive, while NonProf, Wellbeing, Income, Saving, and HHIncome are negative; the Cohabit interval narrowly includes zero in the final production run. With clerical workers as the reference group, most occupational contrasts are modest and readily interpretable.

Model 3 shows that Comput is credibly positive before ADTFI is included and becomes smaller in Model 2. This attenuation is compatible with an ADTFI-related coefficient component, but not proof of a temporal mechanism. Other associations, including QuitJob and HHIncome, change little.

### 5.3 Covariate-adjusted association decomposition

**Figure 2:**
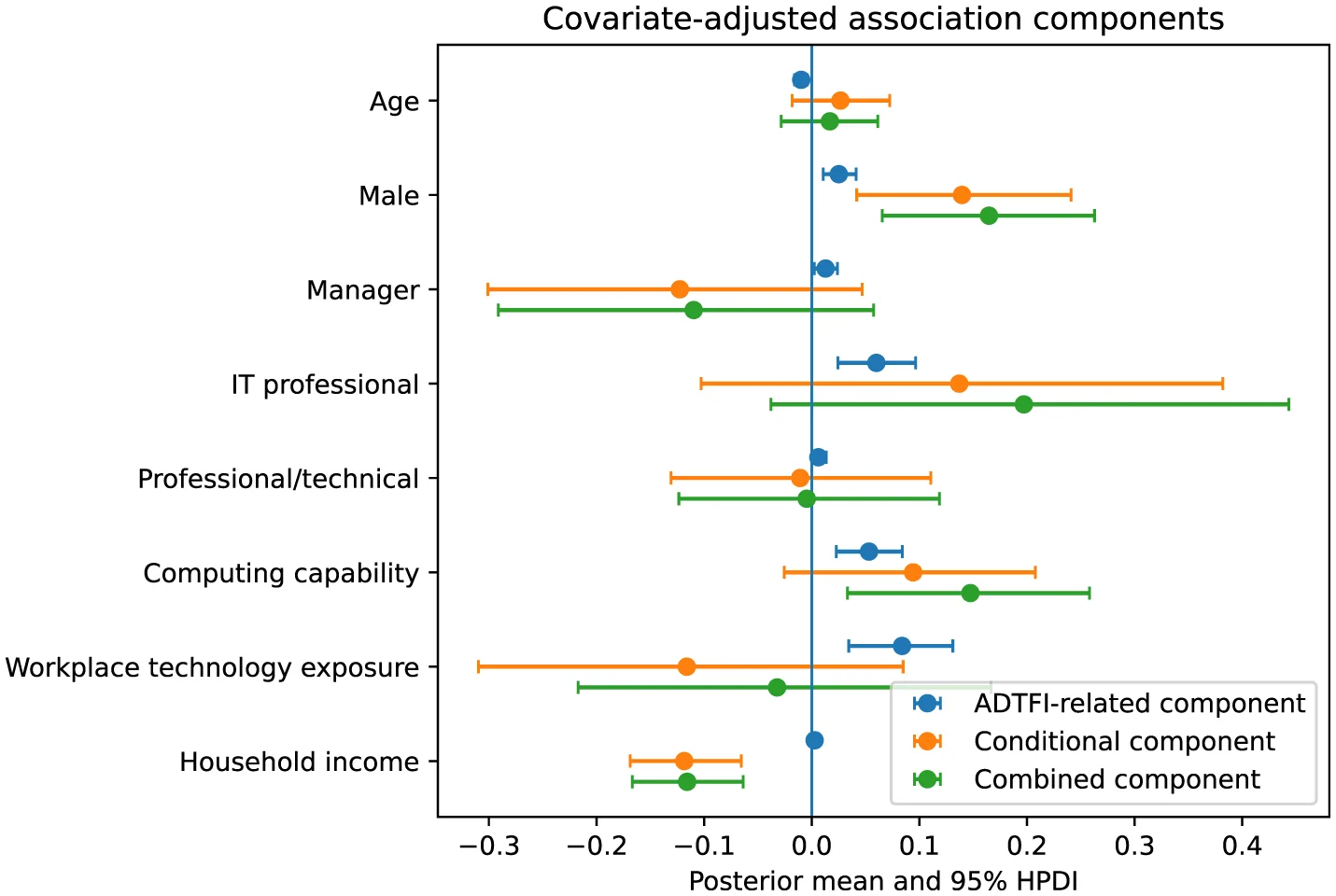
Covariate-adjusted association components for selected variables. Bars are 95% HPDIs.

The decomposition in Table 2 is descriptive. Figure 2 visualizes the selected decomposition results reported in Table 3. For readability, Table 2 reports variables spanning demographic, occupational, digital-environment, and household-resource domains; Tables 3–5 report all covariates. Computing capability has a positive ADTFI-related component, whereas HHIncome has an essentially zero ADTFI-related component and a negative conditional component. TechAdop has a positive ADTFI-related component and an imprecise negative conditional component; the combined component is not credibly different from zero in the audited specification. The data therefore do not support a credibly negative combined TechAdop component.

**Table 2:** Covariate-adjusted coefficient decomposition for selected variables.

Variable	ADTFI-rel.	Conditional	Combined	Pr(*A*^(*F*)^>0)
Age	−0.010 [−0.016, −0.004]	0.027 [−0.018, 0.073]	0.017 [−0.028, 0.061]	0.000
Male	0.025 [0.011, 0.041]	0.140 [0.042, 0.241]	0.165 [0.066, 0.263]	1.000
Manager	0.013 [0.003, 0.024]	−0.123 [-0.301, 0.047]	−0.110 [−0.291, 0.057]	0.999
ITProf	0.060 [0.024, 0.097]	0.137 [−0.103, 0.382]	0.197 [−0.038, 0.443]	1.000
Tech	0.006 [0.000, 0.013]	−0.011 [−0.131, 0.111]	−0.005 [−0.123, 0.119]	0.981
Comput	0.053 [0.023, 0.084]	0.094 [−0.026, 0.208]	0.147 [0.033, 0.258]	1.000
TechAdop	0.084 [0.034, 0.131]	−0.116 [−0.310, 0.085]	−0.032 [−0.217, 0.167]	1.000
HHIncome	0.003 [−0.000, 0.006]	−0.118 [−0.169, −0.066]	−0.116 [−0.167, −0.064]	0.984

Entries are posterior means with 95% HPDIs. The ADTFI and outcome equations were fitted separately. Equal-sized independent random samples from the two posterior distributions were paired to propagate uncertainty under the maintained independence assumption; the components are descriptive coefficient decompositions, not causal mediation effects.

**Table 3:** Model 1: Hierarchical Bayesian panel estimates for ADTFI.

Variable	Mean	95% HPDI
Year22	0.019	[−0.002, 0.040]
Year23	−0.019	[−0.041, 0.003]
**Age**	**−0.097**	**[−0.119**, **−0.074]**
AgeSq	−0.005	[−0.022, 0.013]
Married	0.017	[−0.034, 0.069]
**Male**	**0.248**	**[0.196, 0.299]**
Cohabit	−0.031	[−0.067, 0.006]
WorkStu	0.153	[−0.028, 0.338]
WorkHom	−0.013	[−0.063, 0.034]
**OtherOcc**	**−0.383**	**[−0.671**, **−0.078]**
**Agric**	**−0.151**	**[−0.287**, **−0.018]**
Sales	−0.027	[−0.087, 0.036]
Service	−0.031	[−0.088, 0.025]
**Manager**	**0.127**	**[0.047, 0.204]**
**Trans**	**−0.146**	**[−0.238**, **−0.059]**
**Manuf**	**−0.129**	**[−0.190**, **−0.065]**
**ITProf**	**0.592**	**[0.474, 0.710]**
**Tech**	**0.061**	**[0.004, 0.117]**
Secur	−0.108	[−0.254, 0.043]
**Private**	**0.062**	**[0.019, 0.103]**
NonProf	0.013	[−0.046, 0.073]
Public	0.016	[−0.066, 0.099]
QuitJob	−0.009	[−0.047, 0.029]
**TechAdop**	**0.828**	**[0.740, 0.912]**
**Comput**	**0.525**	**[0.472, 0.578]**
Wellbeing	0.002	[−0.006, 0.009]
Alcohol	0.002	[−0.033, 0.035]
Smoking	−0.019	[−0.066, 0.030]
**Exercise**	**0.033**	**[0.002, 0.063]**
Income	0.004	[−0.003, 0.011]
**Saving**	**0.003**	**[0.001, 0.006]**
**Asset**	**0.006**	**[0.003, 0.009]**
**HHIncome**	**0.026**	**[0.002, 0.051]**
**Urban**	**−0.081**	**[−0.125**, **−0.034]**
**Rural**	**−0.107**	**[−0.184**, **−0.023]**

Variables in bold have 95% HPDIs that exclude zero. Clerical workers are the occupational reference group.

**Table 4:** Model 2: Workplace AI role openness with ADTFI.

Variable	Mean	95% HPDI
Year22	0.029	[−0.022, 0.080]
Year23	0.050	[−0.004, 0.101]
Age	0.027	[−0.018, 0.073]
**AgeSq**	**0.079**	**[0.044, 0.114]**
Married	−0.003	[−0.104, 0.097]
**Male**	**0.140**	**[0.042, 0.241]**
Cohabit	−0.078	[−0.155, 0.001]
WorkStu	−0.121	[−0.539, 0.267]
**WorkHom**	**0.121**	**[0.015, 0.227]**
OtherOcc	−0.567	[−1.445, 0.348]
**Agric**	**0.299**	**[0.032, 0.578]**
Sales	−0.109	[−0.236, 0.018]
Service	−0.029	[−0.150, 0.087]
Manager	−0.123	[−0.301, 0.047]
Trans	0.021	[−0.168, 0.220]
Manuf	−0.060	[−0.196, 0.074]
ITProf	0.137	[−0.103, 0.382]
Tech	−0.011	[−0.131, 0.111]
Secur	−0.204	[−0.511, 0.111]
Private	−0.051	[−0.143, 0.039]
**NonProf**	**−0.162**	**[−0.290**, **−0.035]**
Public	−0.131	[−0.292, 0.041]
**QuitJob**	**0.182**	**[0.094, 0.268]**
TechAdop	−0.116	[−0.310, 0.085]
Comput	0.094	[−0.026, 0.208]
**Wellbeing**	**−0.020**	**[−0.035**, **−0.004]**
Alcohol	0.010	[−0.062, 0.085]
Smoking	−0.078	[−0.177, 0.021]
Exercise	0.016	[−0.057, 0.084]
**Income**	**−0.018**	**[−0.034**, **−0.000]**
**Saving**	**−0.006**	**[−0.011**, **−0.000]**
Asset	0.003	[−0.004, 0.008]
**HHIncome**	**−0.118**	**[−0.169**, **−0.066]**
Urban	−0.011	[−0.095, 0.077]
Rural	−0.024	[−0.176, 0.128]
**ADTFI**	**0.101**	**[0.043, 0.157]**

Variables in bold have 95% HPDIs that exclude zero. Clerical workers are the occupational reference group; ADTFI is mean-centered in estimation.

**Table 5:** Model 3: Workplace AI role openness without ADTFI.

Variable	Mean	95% HPDI
Year22	0.031	[−0.019, 0.083]
Year23	0.048	[−0.005, 0.100]
Age	0.017	[−0.027, 0.063]
**AgeSq**	**0.078**	**[0.043, 0.111]**
Married	0.001	[−0.100, 0.104]
**Male**	**0.162**	**[0.062, 0.258]**
**Cohabit**	**−0.083**	**[−0.161**, **−0.008]**
WorkStu	−0.098	[−0.516, 0.302]
**WorkHom**	**0.119**	**[0.012, 0.222]**
OtherOcc	−0.597	[−1.549, 0.310]
**Agric**	**0.293**	**[0.041, 0.570]**
Sales	−0.107	[−0.235, 0.021]
Service	−0.025	[−0.142, 0.093]
Manager	−0.106	[−0.277, 0.072]
Trans	0.012	[−0.178, 0.206]
Manuf	−0.067	[−0.197, 0.064]
ITProf	0.204	[−0.038, 0.451]
Tech	−0.000	[−0.120, 0.123]
Secur	−0.209	[−0.506, 0.088]
Private	−0.047	[−0.139, 0.044]
**NonProf**	**−0.161**	**[−0.287**, **−0.031]**
Public	−0.126	[−0.296, 0.029]
**QuitJob**	**0.180**	**[0.094, 0.265]**
TechAdop	−0.014	[−0.207, 0.176]
**Comput**	**0.151**	**[0.039, 0.266]**
**Wellbeing**	**−0.020**	**[−0.036**, **−0.005]**
Alcohol	0.010	[−0.065, 0.080]
Smoking	−0.081	[−0.178, 0.017]
Exercise	0.018	[−0.049, 0.088]
**Income**	**−0.018**	**[−0.034**, **−0.000]**
**Saving**	**−0.005**	**[−0.011**, **−0.000]**
Asset	0.004	[−0.002, 0.010]
**HHIncome**	**−0.116**	**[−0.167**, **−0.065]**
Urban	−0.018	[−0.106, 0.071]
Rural	−0.032	[−0.200, 0.119]

Variables in bold have 95% HPDIs that exclude zero. Clerical workers are the occupational reference group.

### 5.4 Lagged specification

The lagged model is elevated to the main results because it partially addresses temporal ordering. The lagged-model estimates are reported in Table 6. Prior ADTFI remains positively associated with subsequent AIAccept after prior AIAccept is controlled, but its magnitude is small relative to the autoregressive term. The posterior random-intercept scale is small [0.033, 95% HPDI [0.000, 0.082]], suggesting that prior AIAccept absorbs most remaining stable between-person heterogeneity. The result supports persistence of an association, not a training effect or causal adaptation process.

**Table 6:** Lagged workplace AI role openness sensitivity (Model 4).

Variable	Mean	95% HPDI
**Lagged ADTFI**	**0.069**	**[0.011, 0.124]**
**Lagged AIAccept**	**0.465**	**[0.438, 0.493]**
TechAdop	−0.050	[−0.256, 0.154]
Comput	0.046	[−0.072, 0.162]
Male	0.072	[−0.009, 0.156]
**HHIncome**	**−0.084**	**[−0.139**, **−0.031]**

Variables in bold have 95% HPDIs that exclude zero. Current-wave AIAccept is regressed on prior-wave ADTFI and prior-wave AIAccept, with the same covariates and 2022 as the transition-year reference. The model includes an individual random intercept and uses 3,915 person-wave observations from 1,964 respondents. The identical focal coefficients are reported in Supplementary Table S10.

### 5.5 Role-specific outcomes

**Figure 3:**
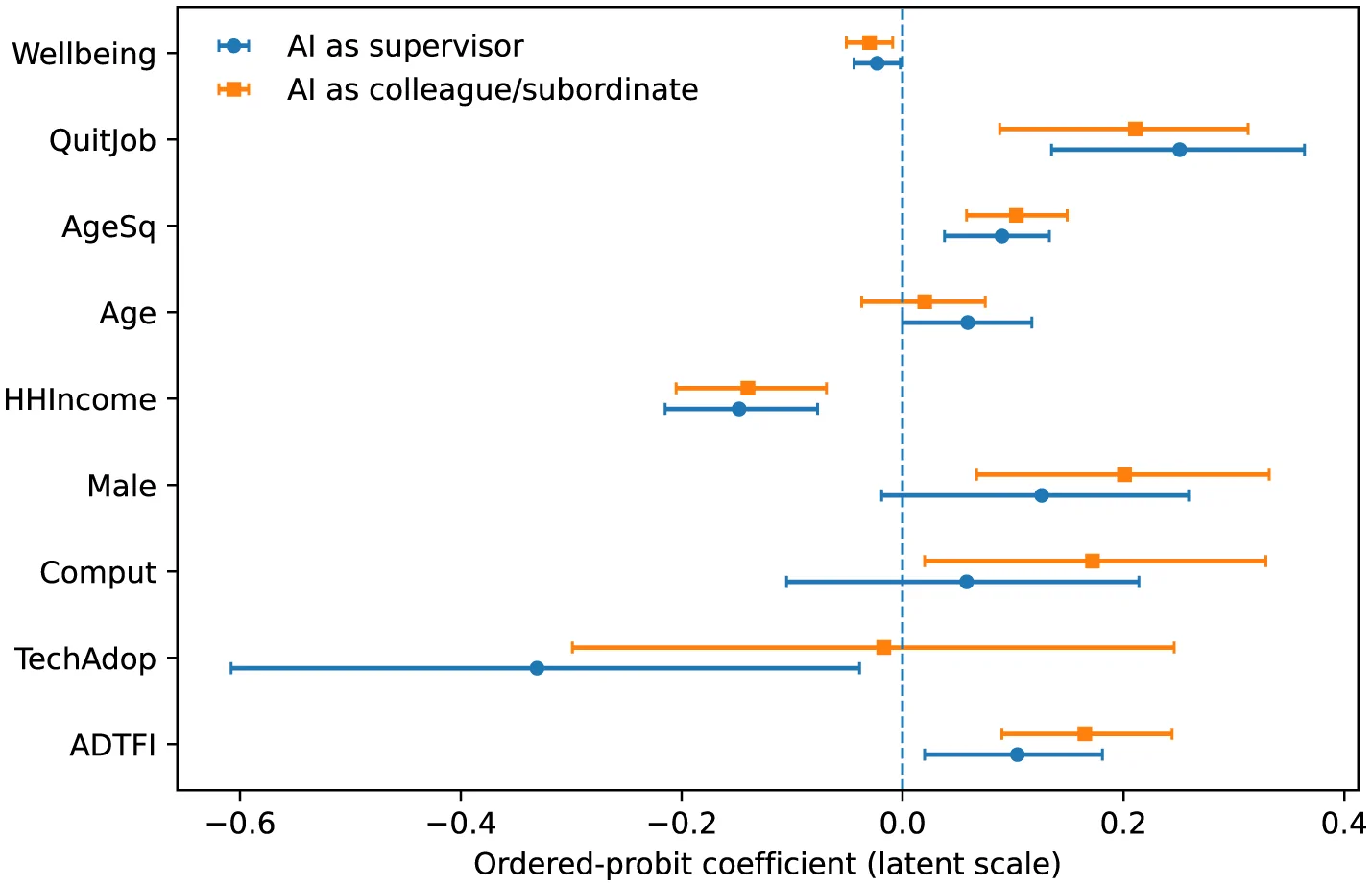
Role-specific ordered-probit associations for selected variables. Bars are 95% HPDIs on the latent standard-normal scale.

The combined AIAccept index pools distinct role relationships. Table 7 reports separate ordered-probit models and Figure 3 compares selected estimates from the two role-specific models. ADTFI is positively associated with openness in both scenarios. In the separately estimated models, the TechAdop coefficient is negative with an interval excluding zero for AI as supervisor, whereas the colleague/subordinate estimate is near zero and imprecisely estimated. Because the models were fitted separately, this pattern is interpreted descriptively and not as a formal test that the TechAdop coefficients differ across roles. Concerns about automated authority are one possible explanation, but surveillance, control, threat, and fairness were not measured and remain alternatives.

**Table 7:** Role-specific Bayesian ordered-probit estimates.

Variable	AI as supervisor	AI as colleague/subordinate
ADTFI	0.104 [0.020, 0.181]	0.165 [0.090, 0.244]
TechAdop	−0.331 [−0.608, −0.039]	−0.017 [−0.299, 0.246]
Comput	0.058 [−0.105, 0.214]	0.172 [0.020, 0.329]
Male	0.126 [−0.019, 0.259]	0.201 [0.067, 0.332]
HHIncome	−0.148 [−0.215, −0.077]	−0.140 [−0.205, −0.069]
Age	0.059 [−0.000, 0.117]	0.020 [−0.037, 0.075]
AgeSq	0.090 [0.038, 0.133]	0.103 [0.058, 0.149]
QuitJob	0.251 [0.135, 0.364]	0.211 [0.088, 0.313]
Wellbeing	−0.023 [−0.044, −0.002]	−0.030 [−0.051, −0.009]

Posterior means with 95% HPDIs on the latent standard-normal scale. Each model uses 5,889 person-wave observations from 1,970 respondents. The items are estimated in separate models because supervisory authority and collaborative or subordinate roles are substantively distinct; no formal cross-role coefficient-difference test is reported.

A direct functional-form sensitivity treats the nine possible values of the combined AIAccept index (1, 1.5, …, 5) as ordered categories. The cumulative-probit model uses 5,888 person-wave observations from 1,970 respondents and includes the full Model 2 covariate set. ADTFI remains positively associated with openness [0.135; respondent-clustered 95% CI [0.070, 0.201]]. TechAdop remains negative but imprecise [−0.125; 95% CI [−0.343, 0.093]], while HHIncome remains negative [−0.134; 95% CI [−0.201, −0.067]]. The focal sign pattern is therefore preserved under a bounded ordinal link. This Supplementary model uses respondent-clustered covariance rather than the full Bayesian random-intercept hierarchy and is interpreted as a functional-form sensitivity, not as a replacement for the panel model (Supplementary Table S14).

### 5.6 Measurement and within–between sensitivities

Using the AI-neutral familiarity index yields a positive coefficient of 0.086 [95% HPDI [0.036, 0.138]], indicating that the main association is not solely produced by including AI, machine-learning, and deep-learning items. The Mundlak sensitivity analysis reported in Supplementary Table S11 shows a positive between-person component and a near-zero within-person component. Digitally familiar employees are generally more open to workplace AI roles, but the data do not show that short-run increases in an individual's familiarity produce corresponding increases in openness.

The formal multiple-imputation sensitivity gives an ADTFI coefficient of 0.108 [Rubin-pooled 95% interval [0.051, 0.165]], close to both the 1NN baseline estimate of 0.101 and the strict complete-case estimate of 0.115. The positive Male and QuitJob associations and the negative HHIncome and Wellbeing associations are also retained. TechAdop remains negative but imprecise, while Comput remains positive with an interval that narrowly includes zero; the larger complete-case Comput coefficient therefore indicates greater sensitivity for this secondary association. Taken together, the focal ADTFI result is not materially dependent on the original 1NN procedure, although some secondary coefficient magnitudes vary with the missing-data strategy (Supplementary Table S7).

## 6 Discussion

The results support a narrower conclusion than a causal mediation account. Advanced digital technology familiarity is a modest correlate of workplace AI role openness, and selected covariate associations can be decomposed algebraically into ADTFI-related and conditional components. The relationship is primarily between persons and persists, at a smaller magnitude, in a lagged model.

The role configuration is theoretically and managerially important. In the separately estimated role-specific models, technology exposure has a negative coefficient with an interval excluding zero for AI as supervisor, whereas the colleague/subordinate estimate is near zero and imprecise. This descriptive contrast is not a formal cross-role coefficient test. One possible explanation is that employees with greater exposure have more realistic knowledge of implementation limitations or react differently when technology is assigned authority. Other possibilities include occupational composition, restructuring, surveillance concerns, role ambiguity, or suppression among correlated digital variables. The data do not adjudicate among these mechanisms.

The practical implications should not be read as support for selecting only already digitally familiar employees. Such a strategy could reproduce existing digital inequality and the study does not show that selection improves implementation outcomes. More defensible implications are to differentiate rollout by role configuration, provide inclusive support and opportunities to acquire relevant knowledge, involve employees in role clarification, and avoid framing lower familiarity as an individual deficit. Because the within-person association is not credibly positive, the study cannot promise that short training interventions will change attitudes; training may nevertheless improve operational competence or reduce uncertainty through mechanisms not identified here.

Several limitations define the scope. ADTFI is subjective familiarity, not performance-tested capability, and both focal constructs may be affected by common-method variance, social desirability, overconfidence, anchoring, and stable response styles. The 13.1% out-of-range prediction rate is substantial enough that the Gaussian AIAccept likelihood should not be interpreted as an adequate model of the full bounded response distribution. We retain the Gaussian index specification only for covariate-adjusted conditional-mean comparisons and the linear coefficient decomposition. The combined-index cumulative-probit and role-specific ordered-probit analyses are therefore treated as essential distributional sensitivities, but they do not reproduce or fully validate the Bayesian random-intercept Gaussian model. The second role item combines AI as a colleague and AI as a subordinate, so the analysis cannot determine whether associations differ between lateral collaboration and human-supervised AI. Education, innovativeness, autonomy, prior technology experience, trust, fairness, surveillance concerns, and replacement anxiety are incompletely observed. The equations are fitted separately, and the baseline model does not estimate cross-equation covariance between their random intercepts. The balanced panel selects stable workers; retained respondents have somewhat higher income and computing capability than excluded respondents, so findings should not be generalized to precarious workers, people leaving employment, or the entire Japanese workforce. The observed-covariate IPW sensitivity supports the focal association but cannot remove selection caused by unobserved employment instability, health, or displacement risk. The original harmonization used single-neighbor imputation across the full variable matrix, including a small number of focal-item values, and therefore treated those completed values as known in the baseline analysis. The formal 20-dataset multiple-imputation sensitivity propagates imputation uncertainty and broadly supports the focal ADTFI result. It remains a pragmatic stochastic chained-equations analysis rather than a jointly estimated multilevel Bayesian missing-data model, and the complete-case comparison indicates that some secondary coefficient magnitudes remain sensitive to the missing-data strategy. Finally, the short panel primarily identifies stable between-person differences.

## 7 Conclusion

Among continuously employed Japanese panel respondents, greater self-reported familiarity with advanced digital technologies is associated with greater openness to hypothetical workplace AI roles. The association is modest, mainly between persons, and differs by role configuration. The study therefore contributes evidence on employee attitudes toward AI as supervisor or colleague/subordinate, not on realized AI adoption or the causal effects of capability-building. Inclusive organizational responses should combine role-sensitive implementation, employee participation, and differentiated support while avoiding selection practices that intensify existing digital inequalities.

## Data Availability

The data analyzed in this study are available from the Panel Data Research Center at Keio University upon application and subject to the Center's eligibility and data-use conditions. Information on the JHPS/KHPS datasets and access procedures is available at https://www.pdrc.keio.ac.jp/en/paneldata/datasets/jhpskhps/.
