# Supplementary material for: Attitudinal openness to AI as supervisor or colleague/subordinate in Japan: the role of advanced digital technology familiarity
Source: Front Artif Intell. 2026 Aug 18;9:1920376. doi: 10.3389/frai.2026.1920376 (PMC13529757; doi:10.3389/frai.2026.1920376)
Supplement: Supplementary file 1 [file Supplementary_file_1.pdf]

Supplementary Material: Attitudinal Openness to AI as Supervisor or Colleague/Subordinate in Japan: The Role of Advanced Digital Technology Familiarity

Makoto Nakakita [1,*] and Takahiro Hoshino,[1,2]

[1]Center for Advanced Intelligence Project, RIKEN, Tokyo, Japan
[2]Faculty of Economics, Keio University, Tokyo, Japan

Correspondence*:
Corresponding Author
makoto.nakakita@riken.jp

S1 Bayesian Estimation Details

This supplementary section provides the likelihood, prior specification, and full conditional posterior distributions for the hierarchical Bayesian panel models introduced in Section 4. The notation follows the model definitions in the main text. The derivations reported here support Models 1–4. The coefficient decomposition combines posterior draws from the separately estimated familiarity and outcome equations as described in the main text.

The likelihood function for the unified model is given by:

$$p(\boldsymbol{y}|\boldsymbol{Z}, \boldsymbol{\delta}, \sigma^2) \propto (\sigma^2)^{-\frac{NT}{2}} \exp\left[-\frac{1}{2\sigma^2}(\boldsymbol{y} - \boldsymbol{Z}\boldsymbol{\delta})^\top(\boldsymbol{y} - \boldsymbol{Z}\boldsymbol{\delta})\right], \tag{S1}$$

where $\boldsymbol{Z} = [\,\boldsymbol{U}, \boldsymbol{X}\,]$ and $\boldsymbol{\delta} = (\boldsymbol{\alpha}^\top, \boldsymbol{\beta}^\top)^\top$.

The hierarchical priors are specified as:

$$\alpha_i \sim \mathcal{N}(\mu_\alpha, \sigma_\alpha^2), \tag{S2}$$

$$\boldsymbol{\beta} \sim \mathcal{N}(\boldsymbol{\mu}_\beta, \boldsymbol{\Sigma}_\beta), \tag{S3}$$

$$\mu_\alpha \sim \mathcal{N}(\varphi_\alpha, \tau_\alpha^2), \tag{S4}$$

$$\sigma_\alpha \sim \mathcal{C}^+(0, s_\alpha), \tag{S5}$$

$$\sigma \sim \mathcal{C}^+(0, s_\sigma). \tag{S6}$$

To facilitate Gibbs sampling, the half-Cauchy priors are represented using inverse-gamma (IG)

auxiliary parameters:

$$\sigma_\alpha^2 \mid \xi_\alpha \sim \mathcal{IG}\left(\frac{1}{2}, \frac{1}{\xi_\alpha}\right), \quad \xi_\alpha \sim \mathcal{IG}\left(\frac{1}{2}, \frac{1}{s_\alpha^2}\right), \tag{S7}$$

$$\sigma^2 \mid \xi_\sigma \sim \mathcal{IG}\left(\frac{1}{2}, \frac{1}{\xi_\sigma}\right), \quad \xi_\sigma \sim \mathcal{IG}\left(\frac{1}{2}, \frac{1}{s_\sigma^2}\right). \tag{S8}$$

Combining the likelihood Eq. (S1) and the priors, the joint posterior distribution is:

$$p(\boldsymbol{\theta}|\mathcal{D}) \propto p(\boldsymbol{y}|\boldsymbol{Z}, \boldsymbol{\delta}, \sigma^2)\, p(\boldsymbol{\alpha}|\mu_\alpha, \sigma_\alpha^2)\, p(\mu_\alpha)\, p(\sigma_\alpha)\, p(\boldsymbol{\beta})\, p(\sigma),$$

where $\boldsymbol{\theta} = (\boldsymbol{\beta}, \boldsymbol{\alpha}, \mu_\alpha, \sigma_\alpha, \sigma)$ and $\mathcal{D} = (\boldsymbol{y}, \boldsymbol{Z})$.

The full conditional posterior distributions for the Gibbs sampler are:

$$\boldsymbol{\beta} \mid \mathcal{D}, \boldsymbol{\theta}_{-\boldsymbol{\beta}} \sim \mathcal{N}\left(\begin{matrix}(\sigma^{-2}\boldsymbol{X}^\top\boldsymbol{X} + \boldsymbol{\Sigma}_\beta^{-1})^{-1} \\ \times\, (\sigma^{-2}\boldsymbol{X}^\top(\boldsymbol{y} - \boldsymbol{U}\boldsymbol{\alpha}) + \boldsymbol{\Sigma}_\beta^{-1}\boldsymbol{\mu}_\beta),\end{matrix} (\sigma^{-2}\boldsymbol{X}^\top\boldsymbol{X} + \boldsymbol{\Sigma}_\beta^{-1})^{-1}\right), \tag{S9}$$

$$\boldsymbol{\alpha} \mid \mathcal{D}, \boldsymbol{\theta}_{-\boldsymbol{\alpha}} \sim \mathcal{N}\left(\begin{matrix}(\sigma^{-2}\boldsymbol{U}^\top\boldsymbol{U} + \sigma_\alpha^{-2}\boldsymbol{I}_N)^{-1} \\ \times\, (\sigma^{-2}\boldsymbol{U}^\top(\boldsymbol{y} - \boldsymbol{X}\boldsymbol{\beta}) + \sigma_\alpha^{-2}\mu_\alpha\mathbf{1}_N),\end{matrix} (\sigma^{-2}\boldsymbol{U}^\top\boldsymbol{U} + \sigma_\alpha^{-2}\boldsymbol{I}_N)^{-1}\right), \tag{S10}$$

$$\mu_\alpha \mid \mathcal{D}, \boldsymbol{\theta}_{-\mu_\alpha} \sim \mathcal{N}\left(\frac{\sigma_\alpha^{-2}\sum_{i=1}^N \alpha_i + \tau_\alpha^{-2}\varphi_\alpha}{\sigma_\alpha^{-2}N + \tau_\alpha^{-2}}, \frac{1}{\sigma_\alpha^{-2}N + \tau_\alpha^{-2}}\right), \tag{S11}$$

$$\sigma_\alpha^2 \mid \mathcal{D}, \boldsymbol{\theta}_{-\sigma_\alpha}, \xi_\alpha \sim \mathcal{IG}\left(\frac{N+1}{2}, \frac{\sum_{i=1}^N(\alpha_i - \mu_\alpha)^2}{2} + \frac{1}{\xi_\alpha}\right), \tag{S12}$$

$$\xi_\alpha \mid \sigma_\alpha \sim \mathcal{IG}\left(1, \frac{1}{\sigma_\alpha^2} + \frac{1}{s_\alpha^2}\right), \tag{S13}$$

$$\sigma^2 \mid \mathcal{D}, \boldsymbol{\theta}_{-\sigma}, \xi_\sigma \sim \mathcal{IG}\left(\frac{NT+1}{2}, \frac{\sum_{i=1}^N\sum_{t=1}^T(y_{it} - \alpha_i - \boldsymbol{x}_{it}^\top\boldsymbol{\beta})^2}{2} + \frac{1}{\xi_\sigma}\right), \tag{S14}$$

$$\xi_\sigma \mid \sigma \sim \mathcal{IG}\left(1, \frac{1}{\sigma^2} + \frac{1}{s_\sigma^2}\right). \tag{S15}$$

S2 Supplementary Data and Robustness Information

This supplementary section documents sample construction, measurement decisions, reference-category choices, diagnostics, and sensitivity analyses that support the interpretation of the main results. Each table is preceded by a short explanation of the inferential question it addresses.

Table S1: Sample construction and AIAccept item availability

Stage	Person-waves	Individuals
Initial harmonized file	13,507	5,021
After residence restrictions	11,061	4,156
Employed observations	7,576	3,017
Balanced continuously employed panel	5,928	1,976
Post-harmonization outcome sample with both item values on the 1–5 scale	5,888	1,970

The balanced-panel restriction may select respondents with more stable employment and resources. The following baseline comparison describes observed differences between retained and excluded employed respondents; it is descriptive and does not by itself correct selection.

Table S2: Baseline comparison of retained and excluded employed respondents

Variable	Retained mean	Retained SD	Excluded mean	Excluded SD
Age	51.525	12.647	51.307	15.935
Male	0.553	0.497	0.487	0.500
Married	0.701	0.458	0.677	0.468
Individual income	14.594	1.864	13.911	3.144
Household income	15.593	0.727	15.519	0.875
ADTFI	1.978	0.746	1.940	0.725
AIAccept	2.346	1.168	2.399	1.190
TechAdop	0.080	0.172	0.092	0.212
Comput	0.294	0.335	0.276	0.330

Note. Retained respondents are the 1,976 people included in the three-wave continuously employed panel. Excluded respondents are 733 people employed at the 2021 baseline who did not satisfy the three-wave retention rule. Variable-specific raw item availability may produce small differences in the denominator.

To assess sensitivity to observed selection, retention probabilities were estimated from 2021 age, sex, marital status, ADTFI, AIAccept, TechAdop, Comput, income, household income, region, and source panel. Inverse-probability weights were formed after trimming estimated propensities at the first and ninety-ninth percentiles. The weighted clustered regression is a supplementary non-Bayesian sensitivity analysis and cannot adjust for unobserved determinants of retention.

Table S3: Inverse-probability-weighted retention sensitivity for selected Model 2 associations

Variable	Estimate	95% interval
ADTFI	0.149	[0.078, 0.220]
TechAdop	-0.149	[-0.387, 0.089]
Comput	0.105	[-0.035, 0.244]
Male	0.135	[0.028, 0.243]
HHIncome	-0.145	[-0.218, -0.072]

Note. Cluster-robust 95% confidence intervals. The sensitivity is used to assess direction and magnitude under observed-covariate reweighting, not as a substitute for a joint Bayesian selection model.

Table S4: Raw and post-harmonization availability of the two AIAccept items

Data stage	Availability pattern	Person-wave observations
Raw wave files	Both role items observed	5,780
Raw wave files	Exactly one role item observed	7
Raw wave files	Neither role item observed	141
Post-harmonization file	Both item values on the 1–5 scale	5,888
Post-harmonization file	Exactly one value on the 1–5 scale	2
Post-harmonization file	Neither value on the 1–5 scale	38

Note. The difference between the raw and post-harmonization counts reflects the original 1NN procedure. The primary outcome models use the post-harmonization file and require both item values to fall on the valid 1–5 scale. The raw complete-case sensitivity excludes every record with missing focal items or covariates.

The raw-data audit shows that missingness in the focal scale items was low, whereas some monetary covariates had more substantial missingness. The counts below refer to the 5,928 records that match the baseline analytical IDs and waves before imputation.

Table S5: Model inputs with nonzero pre-imputation missingness

Variable or raw item	Missing observations	Missing percentage
AI-supervisor item	143	2.41%
AI colleague/subordinate item	144	2.43%

Continued on next page

Table S5: Model inputs with nonzero pre-imputation missingness (continued)

Variable or raw item	Missing observations	Missing percentage
ADTFI: IoT familiarity	47	0.79%
ADTFI: cloud familiarity	40	0.67%
ADTFI: AI familiarity	38	0.64%
ADTFI: RPA familiarity	54	0.91%
ADTFI: machine-learning familiarity	40	0.67%
ADTFI: deep-learning familiarity	39	0.66%
TechAdop: cloud	92	1.55%
TechAdop: robotics	88	1.48%
TechAdop: RPA	99	1.67%
TechAdop: AI	91	1.54%
TechAdop: wearable devices	95	1.60%
Comput: spreadsheet processing	36	0.61%
Comput: macro processing	37	0.62%
Comput: programming-based processing	36	0.61%
Occupation	54	0.91%
Organization type	65	1.10%
Employment status	1	0.02%
Quit-job item	83	1.40%
Subjective well-being	18	0.30%
Alcohol	41	0.69%
Smoking	40	0.67%
Exercise	40	0.67%
Individual income	310	5.23%
Household savings	418	7.05%
Household assets	463	7.81%
Household income	554	9.35%

Note. Missing-value codes were reconstructed from the original raw wave files using the recoding rules in the supplied harmonization notebook. The table lists every Model 2 input with nonzero pre-imputation missingness; omitted inputs had no missing values in the matched raw records. The original 1NN imputer was applied to the full 93-column matrix rather than to a restricted set of continuous covariates.

A balanced complete-case sensitivity requires every Model 2 input to be observed in all three waves. This is a deliberately strict definition and therefore reduces the sample to 1,158 respondents. The comparison focuses on the coefficients most central to the paper's substantive interpretation.

Table S6: Complete-case sensitivity for selected Model 2 coefficients

Variable	Baseline mean 95% HPDI	Complete-case mean 95% HPDI
ADTFI	0.101 [0.043, 0.157]	0.115 [0.045, 0.189]
TechAdop	-0.116 [-0.310, 0.085]	-0.131 [-0.385, 0.122]
Comput	0.094 [-0.026, 0.208]	0.213 [0.069, 0.382]
Male	0.140 [0.042, 0.241]	0.135 [0.008, 0.262]
HHIncome	-0.118 [-0.169, -0.066]	-0.136 [-0.222, -0.048]
QuitJob	0.182 [0.094, 0.268]	0.227 [0.119, 0.344]
Wellbeing	-0.020 [-0.035, -0.004]	-0.032 [-0.052, -0.012]

Note. The complete-case model uses 3,474 person-wave observations from 1,158 respondents with all Model 2 items and covariates observed in all three waves. It was estimated with four chains, 2,000 warm-up iterations, and 2,000 retained iterations per chain. The focal ADTFI estimate and the principal coefficient directions are robust, although the stricter sample changes some magnitudes.

The comparison below evaluates whether the principal Model 2 associations depend on the original single-neighbor imputation. It contrasts the audited 1NN baseline with the Rubin-pooled multiple-imputation sensitivity and the strict balanced complete-case model. The intervals are reported on their native inferential scales and should be compared primarily for direction, magnitude, and overlap.

Table S7: Sensitivity of focal Model 2 associations to missing-data handling

Variable	1NN baseline	Multiple imputation	Complete case
ADTFI	0.101 [0.043, 0.157]	0.108 [0.051, 0.165]	0.115 [0.045, 0.189]
TechAdop	-0.116 [-0.310, 0.085]	-0.141 [-0.347, 0.064]	-0.131 [-0.385, 0.122]
Comput	0.094 [-0.026, 0.208]	0.108 [-0.012, 0.228]	0.213 [0.069, 0.382]
Male	0.140 [0.042, 0.241]	0.150 [0.049, 0.251]	0.135 [0.008, 0.262]
HHIncome	-0.118 [-0.169, -0.066]	-0.147 [-0.215, -0.079]	-0.136 [-0.222, -0.048]
QuitJob	0.182 [0.094, 0.268]	0.188 [0.101, 0.275]	0.227 [0.119, 0.344]
Wellbeing	-0.020 [-0.035, -0.004]	-0.021 [-0.037, -0.006]	-0.032 [-0.052, -0.012]

Note. The multiple-imputation column combines 20 stochastic chained-equations completed datasets using Rubin's rules. Each completed dataset was estimated with four chains, 3,000 warm-up iterations, and 3,000 retained iterations per chain. AIAccept was not imputed; both raw AI-role items were required, yielding 5,780 person-wave observations from 1,961 respondents. Baseline and complete-case intervals are 95% HPDIs; multiple-imputation intervals are Rubin-pooled 95% intervals.

Table S8: Occupational category counts

Category	Person-waves	Individuals
Unclassified/other code 0	5	4
Agriculture/fishery	146	54
Mining	0	0
Sales	852	347
Service	1,122	468
Managerial	265	140
Clerical (reference)	1,106	444
Transport/communication	263	115
Manufacturing/construction	906	368
IT professional	165	64
Professional/technical	999	417
Security	96	39
Other occupation	3	3

Note. Clerical workers are the reference group in the final models. The previous residual reference combined codes 0, 2, and 12 and contained only eight person-wave observations.

The AI-neutral sensitivity reduces conceptual overlap between advanced-technology familiarity

and the AI-role outcome. It is emphasized in the main text because the full index contains three AI-related labels.

Table S9: AI-neutral familiarity-index sensitivity

Variable	Mean	95% HPDI
**AI-neutral familiarity index**	**0.086**	**[0.036, 0.138]**
TechAdop	-0.121	[-0.322, 0.090]
Comput	0.097	[-0.020, 0.218]
**Male**	**0.138**	**[0.043, 0.238]**
**HHIncome**	**-0.117**	**[-0.171, -0.065]**

Note. The alternative index contains IoT, cloud, and RPA only; AI, machine learning, and deep learning are excluded.

The following table duplicates the final lagged values shown in the main text so that there is a single internally consistent numerical record.

Table S10: Full lagged workplace AI role openness sensitivity

Variable	Mean	95% HPDI
**Lagged ADTFI**	**0.069**	**[0.011, 0.124]**
**Lagged AIAccept**	**0.465**	**[0.438, 0.493]**
TechAdop	-0.050	[-0.256, 0.154]
Comput	0.046	[-0.072, 0.162]
Male	0.072	[-0.009, 0.156]
**HHIncome**	**-0.084**	**[-0.139, -0.031]**

Note. Current-wave AIAccept is regressed on prior-wave ADTFI and prior-wave AIAccept, with the same covariates and 2022 as the transition-year reference. The model includes an individual random intercept; its posterior scale is 0.033 [0.000, 0.082]. The model uses 3,915 person-wave observations from 1,964 respondents.

The Mundlak analysis evaluates whether the focal association reflects short-run within-person movement or stable differences across respondents.

Table S11: Mundlak-style within–between sensitivity for focal variables

Component	Estimate	Lower	Upper
Within-person ADTFI deviation	0.024	-0.053	0.102
Between-person mean ADTFI	0.176	0.081	0.271
Within-person TechAdop deviation	-0.126	-0.399	0.147
Between-person mean TechAdop	-0.181	-0.508	0.147

Note. Cluster-robust correlated-random-effects sensitivity analysis. It is supplementary to, rather than part of, the main Bayesian sampler.

The final production runs were audited at the model level before the numerical tables were regenerated. All substantive and scale parameters in Models 1–4 met the prespecified convergence thresholds, including the small random-intercept scale in the lagged model.

Table S12: MCMC diagnostics for the final production models

Specification	Warm-up/ retained	Max $\hat{R}$	Min bulk ESS	Min tail ESS
Model 1: ADTFI	4,000/4,000	1.003	1,850	3,123
Model 2: AIAccept with ADTFI	2,500/2,500	1.003	1,399	2,379
Model 3: AIAccept without ADTFI	2,000/2,000	1.006	1,101	1,906
Model 4: lagged outcome	3,000/3,000	1.001	1,914	2,387

Note. All specifications use four chains. The maxima and minima are computed across every reported slope, intercept, and scale parameter in each final production run. Model 4 retains an individual random intercept; ASIS interweaving stabilized sampling of its small scale parameter.

The Gaussian models reproduce central moments but generate predictions outside the bounded response scale. In particular, the 13.1% out-of-range rate for AIAccept is substantial and shows that the Gaussian likelihood is not an adequate model of the full response distribution. We retain the Gaussian specification only for conditional-mean comparisons and the transparent linear decomposition. The ordinal sensitivities below are therefore treated as essential checks of the focal sign pattern, while not constituting a complete validation or reproduction of the hierarchical Gaussian model.

Table S13: Posterior-predictive calibration of the Gaussian index models

Outcome	Observed mean	Observed SD	Predicted mean	Predicted SD	Predicted outside 1–5
ADTFI	1.979	0.756	1.977	0.712	7.6%
AIAccept	2.377	1.160	2.373	1.164	13.1%

Note. Predictions integrate over the estimated random-intercept distribution. The 13.1% rate for AIAccept is treated as evidence of an imperfect Gaussian approximation rather than as a negligible discrepancy.

Table S14: Nine-category cumulative-probit sensitivity for combined AIAccept

Variable	Estimate	95% cluster-robust CI
Year23	0.051	[0.003, 0.099]
Age	0.020	[-0.023, 0.063]
AgeSq	0.061	[0.027, 0.094]
Male	0.118	[0.020, 0.216]
QuitJob	0.249	[0.150, 0.348]
TechAdop	-0.125	[-0.343, 0.093]
Comput	0.099	[-0.030, 0.227]
Wellbeing	-0.016	[-0.035, 0.002]
HHIncome	-0.134	[-0.201, -0.067]
ADTFI	0.135	[0.070, 0.201]

Note. The combined AIAccept index has nine possible ordered values (1, 1.5, . . . , 5), which are mapped to nine ordered categories. The model uses 5,888 person-wave observations from 1,970 respondents and includes all baseline Model 2 covariates; selected coefficients are shown. Standard errors and confidence intervals are clustered by respondent. This cumulative-probit model is a functional-form sensitivity and does not reproduce the full Bayesian random-intercept hierarchy.

Table S15: Full role-specific Bayesian ordered-probit estimates

Variable	AI as supervisor	AI as colleague/ subordinate
ADTFI	0.104 [0.020, 0.181]	0.165 [0.090, 0.244]
TechAdop	-0.331 [-0.608, -0.039]	-0.017 [-0.299, 0.246]
Comput	0.058 [-0.105, 0.214]	0.172 [0.020, 0.329]
Male	0.126 [-0.019, 0.259]	0.201 [0.067, 0.332]
HHIncome	-0.148 [-0.215, -0.077]	-0.140 [-0.205, -0.069]
Age	0.059 [-0.000, 0.117]	0.020 [-0.037, 0.075]
AgeSq	0.090 [0.038, 0.133]	0.103 [0.058, 0.149]
QuitJob	0.251 [0.135, 0.364]	0.211 [0.088, 0.313]
Wellbeing	-0.023 [-0.044, -0.002]	-0.030 [-0.051, -0.009]

Note. Posterior means with 95% HPDIs on the latent standard-normal scale. Each model uses 5,889 person-wave observations from 1,970 respondents. The items are estimated in separate models because supervisory authority and collaborative or subordinate roles are substantively distinct; no formal cross-role coefficient-difference test is reported.

S3 Original Survey Wording and Translation Note

The questionnaire extracts below document the content of ADTFI and AIAccept. The supplied form is the married-respondent version; the focal modules are reproduced to document item wording, while the analytical sample is defined by employment continuity and item availability rather than marital status.

Table S16: Original Japanese and official English wording for the focal items

Construct	Japanese wording	Official English wording
ADTFI stem	「あなたは以下の情報技術について、どれくらいご存知ですか。」	"How much knowledge do you have about the following information technologies?"
ADTFI items	「IoT」「クラウド」「人工知能（AI）」「RPA」「機械学習」「深層学習」	IoT, Cloud, AI, RPA, Machine learning, Deep learning
AIAccept stem	「将来、AIがあなたの上司・同僚・部下となるとしたら、どの程度抵抗感がありますか。」	"To what extent do you feel resistance if AI becomes your boss, colleague, or subordinate in the future?"
AIAccept items	「上司」「同僚・部下」	Boss; Colleague, or subordinate
